# Melanopsin Ganglion Cells: A Different Way of Seeing Things

**DOI:** 10.1371/journal.pbio.1001003

**Published:** 2010-12-07

**Authors:** Caitlin Sedwick

**Affiliations:** Freelance Science Writer, San Diego, California, United States of America

**Figure:**
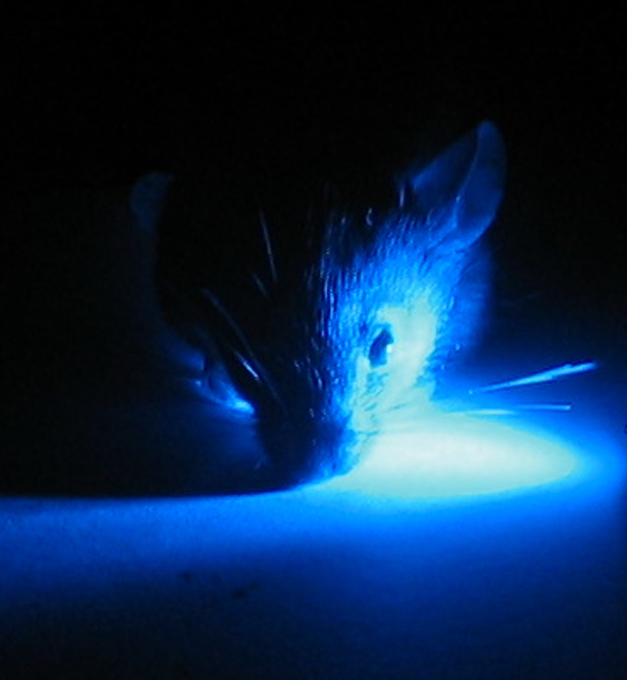
A relatively recently discovered retinal photoreceptor with maximal sensitivity to blue light has previously been thought to influence mostly sub-cortical, reflex light responses. New research shows that it also allows conventional visual centers in the mouse brain to accurately assess brightness.

The major features and functioning of the mammalian visual system are well known. For many years, the consensus has been that incoming light is detected by two specialized cell types—rods and cones—which reside in the retina, the tissue that lines the back of the eye. These cells contain specialized photopigments—proteins that set off a cellular signaling cascade when they are struck by photons of light, generating a signal that is passed through neurons in the retina, along the optic nerve, and finally to the regions of the brain that process visual information.

The photopigment in rods detects dim light (allowing for nighttime vision), while cones express photopigments that respond to brighter light (for daytime vision). But ten years ago, it was discovered that rods and cones aren't the only light-sensing cells in the retina. There is a third kind of cell that expresses a unique photopigment called melanopsin. These cells, called melanopsin retinal ganglion cells (mRGCs), were not thought to be involved in vision. Instead, it was believed that they regulate non-sight–related responses like the pupil constriction reflex and establishment of the circadian cycle. But in this month's issue of *PLoS Biology*, Timothy Brown, Robert Lucas, and colleagues show that mRGCs could actually play an important role in visual perception.

Why did researchers think mRGCs weren't involved in vision? One reason was that previous studies had shown mRGCs did not seem to make contacts with the parts of the brain that process visual signals. Instead, they only seemed to contact brain regions controlling the pupillary constriction reflex and those involved in setting the circadian cycle. However, new research suggests that the methods used to trace mRGC–brain connections might have actually failed to detect a large number of melanopsin-expressing retinal cells. Therefore, Brown et al. revisited this issue, using a new method to trace the targets of mRGC connections in the central nervous system. This new approach showed that many mRGCs actually make connections with the areas of the brain that normally process visual information.

This finding prompted Brown and colleagues to explore whether mRGCs play a role during vision. To do this, they used mice whose retinas lack rods and cones; logically, in these animals, any activity in the visual regions of the brain would have to be due to mRGCs. When these animals' retinas are stimulated with light, mRGCs evoke an intense, prolonged response from neurons in the brain's visual centers—a level of activity only slightly lower than what is observed in animals with intact rods and cones.

Next, the group looked at how mRGCs contribute to vision in animals with intact rods and cones. To separate the output of mRGCs from that of rods and cones, the authors used a genetically engineered mouse whose red-sensitive cone photopigment had been replaced with a human version that responds well to light at the extreme red end of the spectrum. In these animals, a stimulus of intense, extremely red light activates cones and rods but not mRGCs, while intense blue light provokes responses from all three kinds of cells. Therefore, subtracting the brain's red-light responses from its blue-light responses shows the contribution of mRGCs to light perception. These experiments confirmed that mRGCs evoke sustained activity in the visual centers of the brain, while mice lacking melanopsin protein have impaired (although not altogether absent) sustained responses to light.

Collectively, these findings led the authors to conclude that mRGCs inform the brain about the irradiance (that is, brightness or intensity) and protracted presence of a light stimulus that is hitting the retina. This information isn't available from rods and cones because they either become saturated at low light levels (rods) or do not provoke much sustained activity in the brain (cones). The potential for mRGCs to contribute to visual perception may explain why people with conditions or diseases that progressively destroy their rods and cones can often still perceive the presence of ambient light—something that may be important if this finding can be harnessed in clinical or therapeutic settings. In the meantime, vision scientists will have to rework their models of how visual perception works, to integrate mRGCs into the picture.

**Brown TM, Gias C, Hatori M, Keding SR, Semo M, et al. (2010) Melanopsin Contributions to Irradiance Coding in the Thalamo-Cortical Visual System. doi:10.1371/journal.pbio.1000558**

